# The effect of paclitaxel- and fisetin-loaded PBM nanoparticles on apoptosis and reversal of drug resistance gene ABCG2 in ovarian cancer

**DOI:** 10.1186/s13048-023-01308-w

**Published:** 2023-11-21

**Authors:** Melayshia McFadden, Santosh Kumar Singh, Briana Kinnel, Sooryanarayana Varambally, Rajesh Singh

**Affiliations:** 1 Department of Microbiology, Biochemistry, and Immunology, Morehouse School of Medicine, Atlanta, GA 30310 USA; 2 Cancer Health Equity Institute, Morehouse School of Medicine, Atlanta, GA 30310 USA; 3 Department of Pathology, The University of Alabama at Birmingham, Birmingham, AL 35294 USA

**Keywords:** Ovarian cancer, Nanoparticles, Targeted drug delivery, Drug resistance

## Abstract

**Background:**

High-grade serous ovarian cancer (OvCa) is the most common type of epithelial OvCa. It is usually diagnosed in advanced stages, leaving a woman’s chance of survival below 50%. Despite traditional chemotherapeutic therapies, there is often a high recurrence rate following initial treatments. Hence, a targeted drug delivery system is needed to attack the cancer cells and induce apoptosis, overcome acquired drug resistance, and protect normal cells from cytotoxicity. The present study shows that targeting folate receptor alpha (FRα) through planetary ball milling (PBM) nanoparticles (NPs) induces apoptosis in OvCa cells.

**Results:**

Human tissue microarrays (TMAs) show overexpression of FRα in Stage IV OvCa tissues compared to matched normal tissues. They provide a focus for a targeted delivery system. We formulated PBM nanoparticles encapsulated with paclitaxel (PTX) or fisetin (Fis) and conjugated with folic acid (FA). The cytotoxic effect of these PBM NPs reduced the concentration of the toxic chemotherapy drug PTX by five-fold. The combined treatment of PTX-FA NPs and Fis-FA NPs inhibited cell proliferation and induced apoptosis more extensively than the individual drugs alone. Apoptosis of OvCa cells, determined by flow cytometry, showed an increase from 14.4 to 80.4% (OVCAR3 cells) and from 2.69 to 90.0% (CAOV3 cells) in the number of apoptotic cells. Also, expressions of the pro-apoptotic markers, BAK and active caspase-3, were increased after PTX-FA + Fis-FA PBM NP treatment. In addition to looking at targeted treatment effects on apoptosis, drug resistance was investigated. Drug resistance in OvCa cells was reversed by ABCG2, an ABC-transporter marker.

**Conclusions:**

Our study shows that PTX-FA and Fis-FA PBM NPs directly target platinum-resistant OvCa cells, induce cytotoxic/apoptotic effects, and reverse multi-drug resistance (MDR). These findings allow us to create new clinical applications using PTX-FA and Fis-FA combination nanoparticles to treat drug-resistant cancers.

## Background

In 2023, approximately 20,000 women in the United States will receive a new diagnosis of ovarian cancer (OvCa), and more than 13,000 women will die from this disease [1]. The 5-year relative survival rate for all types of OvCa is 49.7%. However, the survival rate relates to the stage, type, and grade of OvCa at the time of diagnosis. There are various types of OvCa, but high-grade serous carcinoma (HGSC) is the most common type of epithelial OvCa, accounting for approximately 70–80% of all OvCas [2–5]. HGSC is usually diagnosed in advanced stages and treated with tumor-debulking surgery and platinum-based chemotherapy; however, a high recurrence rate following the initial treatment has often been observed [6]. Most of these relapsed cases are less curable and have a higher incidence of drug resistance [7]. Hence, there is a need for a drug delivery system that targets the cancer cells, and not normal cells, to induce apoptosis, overcome acquired drug resistance, and protect normal cells from cytotoxicity [8].

OvCa cells overexpress folate receptor alpha (FRα) compared to normal ovarian cells [9, 10]. FRα binds to high-affinity folic acid (FA) and induces cellular processes, such as cell division, proliferation, and metastasis [11]. For FR-targeted therapy, Singh et al. [12, 13] formulated planetary ball milling nanoparticles (PBM-NPs) with FA on the surface. PBM NPs encapsulate drugs and target cancer cells to overcome chemoresistance [14, 15]. PTX, a common chemotherapeutic drug, has a high efficacy for treating primary and relapsed OvCa. However, administration of PTX for relapsed OvCa yields short response rates between 20% and 62%, often leading to toxicity and death [16, 17]. To overcome this toxicity, combining effective chemotherapeutic drugs with non-toxic natural compounds is a recent strategy that can decrease the intrinsic toxicity of anti-cancer agents, decrease the drug side effects, increase the efficacy of treatment, and improve patient compliance [18]. Fisetin (7,3’,4’-flavon-3-ol, Fis) is a naturally occurring fruit and vegetable flavonoid. In cancer research, flavonoids are the most utilized natural compounds because they can reverse MDR by killing resistant cancer cells or causing them to be re-sensitized to anti-cancer drugs [19, 20].

Drug delivery systems have shown remarkable efficacy in diagnosing and treating various cancers. Nanotechnology can enhance drug solubility, stability, and half-life while minimizing off-target effects and concentrating drugs at the target site. Exploiting overexpressed receptors enables targeted delivery of therapeutic nanoparticles into cancer cells, minimizing harm to healthy cells [21]. This is especially important in OvCa, where effective treatment of metastasized ovarian tumors is crucial. Utilizing the concepts that targeted therapy grants direct access to cancer cells and combining effective chemotherapeutic drugs with non-toxic natural compounds increases treatment efficacy, a new therapy for resistant OvCa can be explored. The present study investigated the synergistic cytotoxic/apoptotic effects of PTX-FA and Fis-FA-encapsulated PBM NPs for OvCa cells and assessed their impact on molecular signaling pathways. Our central hypothesis is that if Fis-FA and PTX-FA encapsulated PBM NPs can specifically target FRα on OvCa Cells, then synergistic cytotoxic/apoptotic effects can be induced.

## Results

### FR expression in human OvCa tissue and cells

**Fig. 1:**
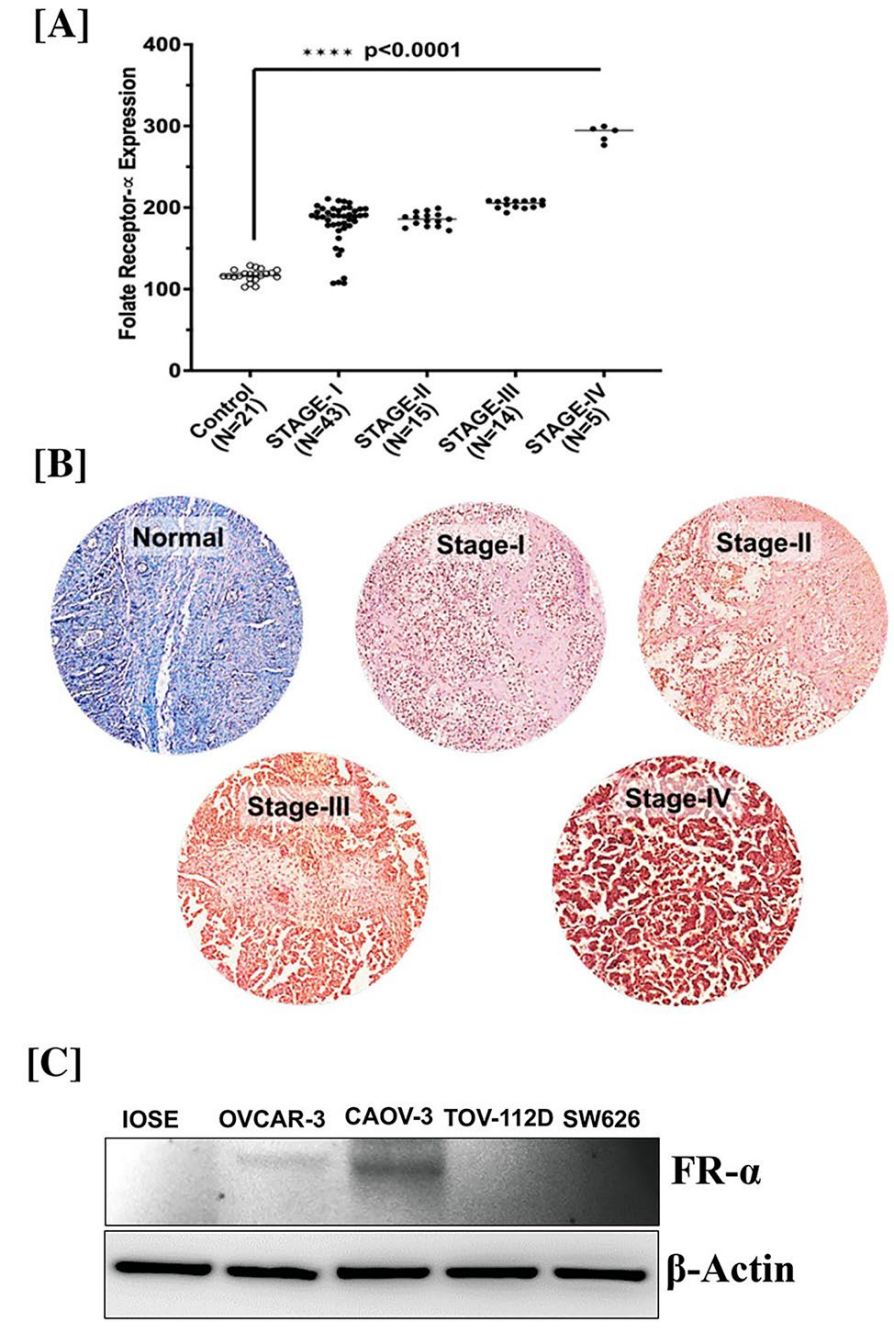
FR Expression in human OvCa tissue and cells. **[A]** Ovarian tissues were stained with anti-FRα antibody and DAB. FRα expression was highest in Stage IV OvCa tissues compared to matched normal tissues. **[B]** An Aperio ScanScope slide scanner system with a 40x objective was used to capture digital images of each tissue. **[C]** Immunoblot expression of the anti-FRα antibody in IOSE, OVCAR-3, CAOV-3, TOV-112D, and SW626 cells. β-Actin antibody was used as a loading control for all the samples

Compared to normal ovarian cells, OvCa cells overexpress FRα, making them an appropriate target for our PBM NPs. Both normal and cancer tissues stained with 3,3′-diaminobenzidine (DAB) and analyzed for FRα showed that FRα expression was highest in Stage IV (N = 5) OvCa tissues compared to matched normal tissues (Fig. 1A/B). A western blot was performed to determine further the expression of FRα in OvCa cells (Fig. 1C). We assessed the expression of the anti-FRα antibody in IOSE, OVCAR-3, CAOV-3, TOV-112D, and SW626 cells. OVCAR-3 and CAOV-3 cells had higher FRα expression than other OvCa cells. These observations demonstrated that membrane-bound FRα is a potential target for FA- PBM NPs.

### Functionalization and characterization of PBM NPs

**Fig. 2:**
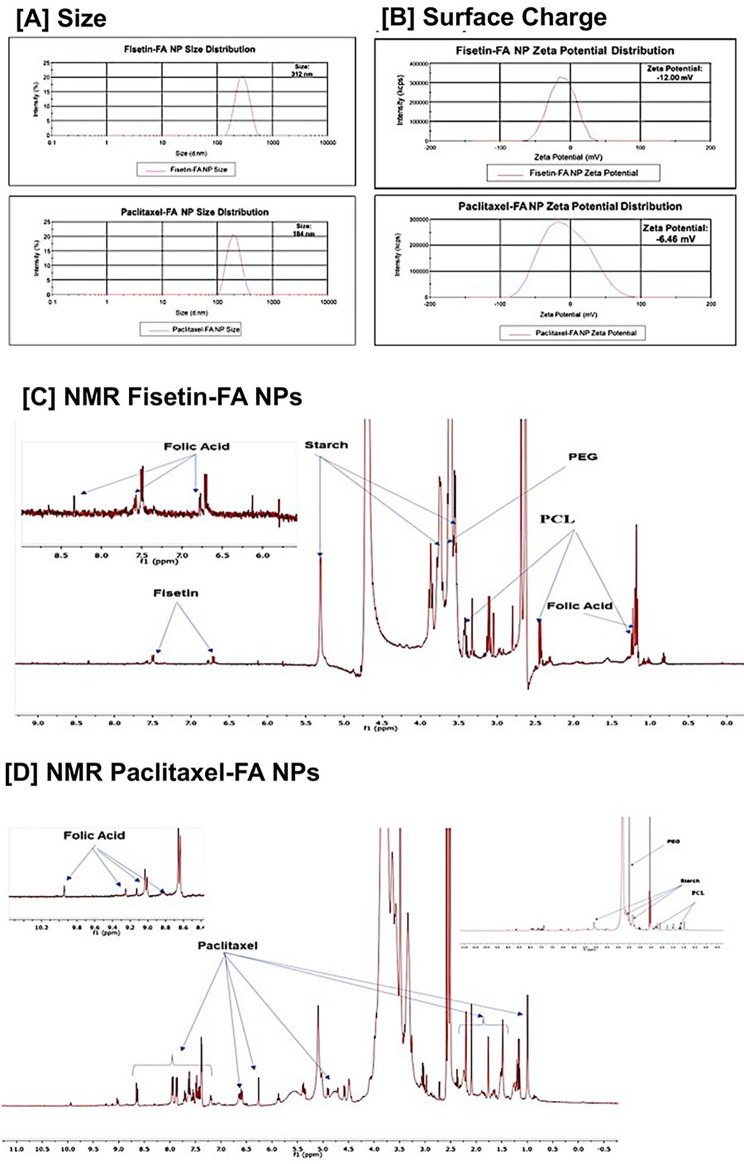
Characterization of OvCa cell specific PBM NPs. Fis- and PTX-encapsulated, FA-conjugated, and PCL-PEG copolymer-coated and uncoated NPs were characterized based on their **[A]** size and **[B]** zeta potential. NMR was conducted to determine the appropriate conjugation of **[C]** Fis-FA-PBM-NPs and **[D]** PTX-FA-PBM NPs, as shown in the 1 H NMR spectra. The arrows indicate peaks in ppm for starch, FA, PEG, polycaprolactone (PCL), Fis, and PTX. A Bruker 400 MHz Spectrometer was used to record the spectra

For effective drug delivery, the functionalization and characterization, i.e., size, zeta potential, and properties after formulation, of NPs must be evaluated. Our results show that after coating, the size of Fis-FA and PTX-FA PBM NPs was 312 nm and 184 nm, respectively (Fig. 2A). Relative to positively charged NPs, NPs with a neutral or negative charge have higher circulating half-lives [22]. This leads to increased accumulation of NPs and encapsulated drugs inside cancers. After coating, the zeta potentials of the Fis-FA PBM NP and PTX-FA PBM NP were − 12.00 mV and − 6.46 mV, respectively (Fig. 2B). The appropriate conjugations of Fis-FA-PBM-NP (Fig. 2C) and PTX-FA-PBM NPs (Fig. 2D) are shown in the 1 H NMR (nuclear magnetic resonance) spectra. These characteristics ensure accurate formulation and promise effective drug delivery.

### Effects of Fis and PTX alone and in combination on cytotoxicity and viability of OvCa cells

To determine the effect of Fis and PTX, alone and in combination, on OVCAR-3 and CAOV-3 cells, cell viability (MTT) assays were performed. The optimal IC_50_ values were calculated for each drug treatment at different time intervals (24, 48, and 72 h) and compared to the control. The cytotoxic effect of all drug treatments was most prominent at 48 h, establishing the following IC50 values for OVCAR3: 73 µM (Fis), 29 µM (Fis-FA NP), 67 nM (PTX), 42 nM (PTX-FA NP), 10 µM Fis/10 nM PTX, and 1 nM PTX-FA NP/5 µM Fis-FA NP. The following IC50 values were determined for CAOV3 cells after 48 h: 35 µM (Fis), 14 µM (Fis-FA NP), 10 nM (PTX), 6 nM (PTX-FA NP), 5 µM Fis/5 nM PTX, and 1 nM PTX-FA NP/ 2 µM Fis-FA NP.

### Combination PBM NPs induce apoptosis in OvCa cells

**Fig. 3:**
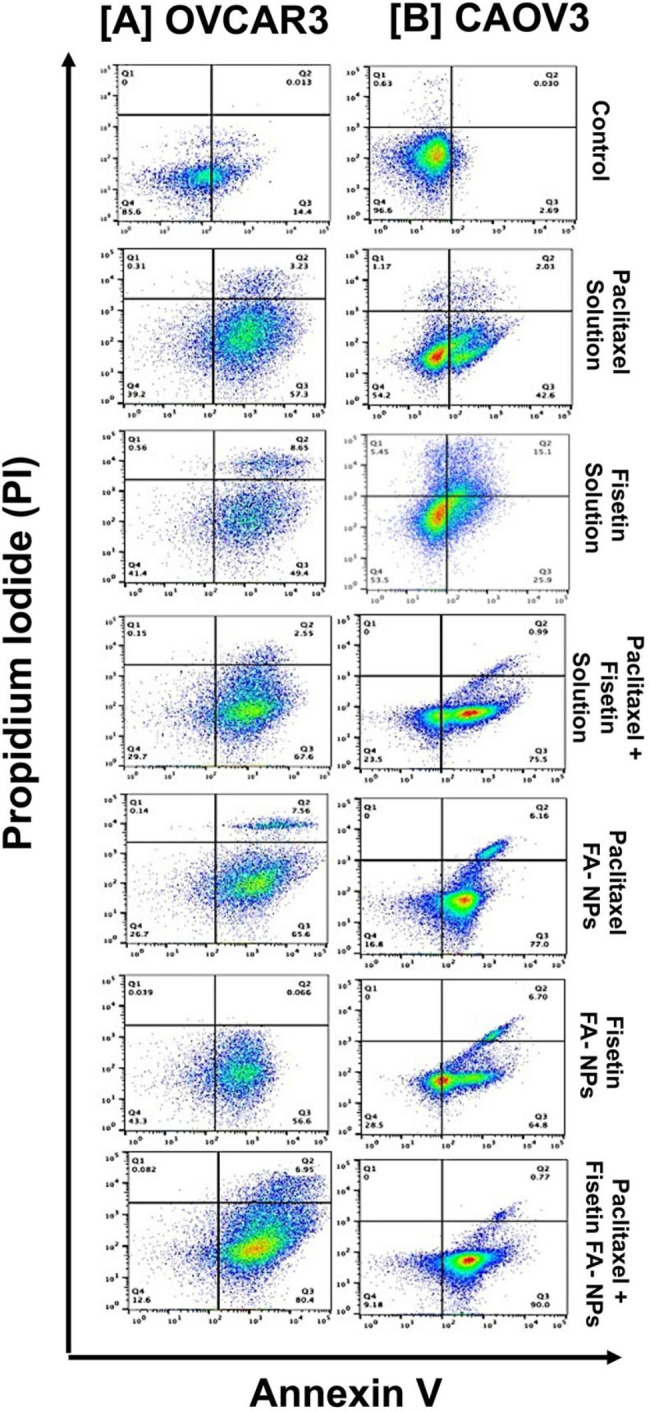
PBM NP-mediated apoptosis in OvCa cells. Flow cytometric analysis of Annexin V-FITC- and PI-stained OvCa cells treated with FA-PBM NPs, **[A]** OVCAR3 cells, and **[B]** CAOV3 cells treated with IC50 concentrations of Fis-FA and PTX-FA NPs, alone and in combination for 48 h. The numbers in each quadrant indicate the percentage of Q1 (necrotic): Annexin (-)/(PI (+), Q2 (late apoptotic): (Annexin (+)/(PI(+), Q3 (viable): Annexin (-)/(PI(-), and Q4 (early apoptotic): Annexin (+)/(PI (-) cells. Data were analyzed by FlowJo software version 10

To determine the effect of combination NPs in OvCa cells, an apoptosis assay was performed that allowed the detection and quantification of cellular events associated with programmed cell death. In Fig. 3 (PI = propidium iodide), data are shown in quadrants Q1, Q2, Q3 and Q4, which represent necrotic (Q1) [Annexin (-)/PI (+)], late apoptotic (Q2) [Annexin (+)/ PI (+)], viable (Q3) [Annexin (-)/PI (-)], and early apoptotic (Q4) [Annexin (+)/PI (-)] cells. In OVCAR-3 cells, apoptosis induced by a combination of Fis-FA- and PTX-FA-NPs was higher (early apoptosis, 80.4%, and late apoptosis, 6.95%) compared to the cells treated with Fis-FA-NPs (early apoptosis, 56.6%, and late apoptosis 0.06%) or PTX-FA-NPs alone (early apoptosis, 65.6%, and late apoptosis,7.56%). Similarly, treatment of CAOV-3 cells with combined Fis-FA-NPs and PTX-FA NPs showed early apoptosis, 90.0%, and late apoptosis, 0.77%, compared to Fis-FA-NPs alone (early apoptosis, 64.8%, and late apoptosis, 6.7%) and PTX-FA-NPs alone (early apoptosis, 77.0%, and late apoptosis, 6.16%). These results demonstrate the anti-cancer role of Fis in combination with PTX when encapsulated in FA-PBM NPs.

### Combined FA-PBM NPs modulate the expression of apoptotic markers in OvCa cells

**Fig. 4:**
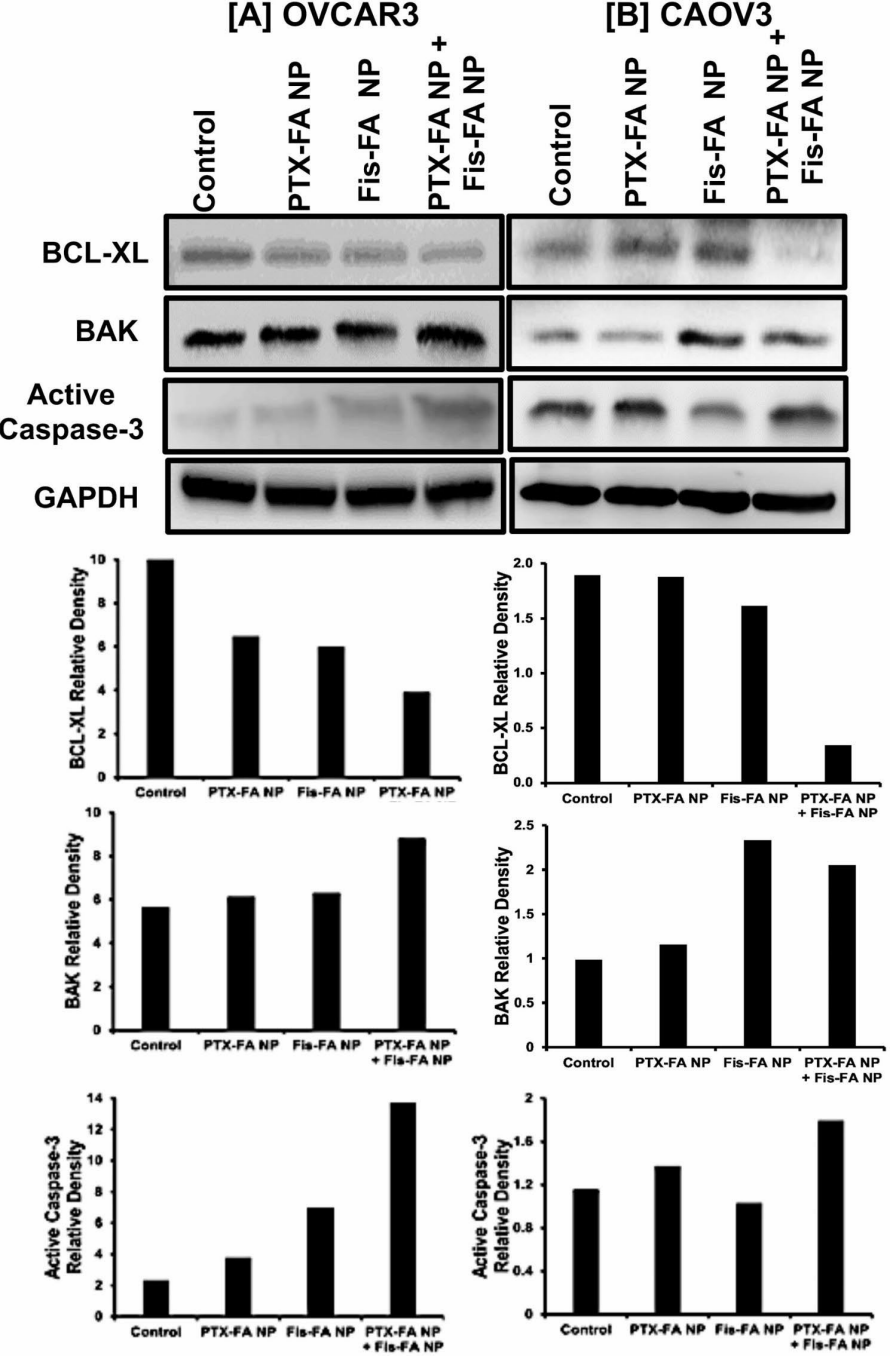
Combined FA PBM NPs modulate the expression of apoptotic markers in OvCa cells. Expression of pro-apoptotic (BAK), apoptotic (active caspase-3), and anti-apoptotic (BCL-XL) proteins in **[A]** OVCAR3 and **[B]** CAOV3 cells treated with Fis and PTX NPs, alone and in combination, for 48 h. An internal standard for equal loading, blots were probed with a GAPDH antibody. BAK, Caspase-3, and BCL-XL band intensity were normalized against GAPDH, and their densitometry are shown below

The effects of Fis and PTX alone, and in combination, on apoptosis were determined by assessing pro-apoptotic, anti-apoptotic, and apoptotic proteins in OvCa cells by immunoblotting. As displayed in Fig. 4, the results show the upregulation of BAK, active caspase-3, and a downregulation of BCL-XL upon treatment compared to control. Interestingly, in CAOV3 cells, the combination NP-treatment was less effective than FA-Fis-NP for BAK expression, indicating the possibility of post-transcriptional changes of BAK protein.

**Fig. 5:**
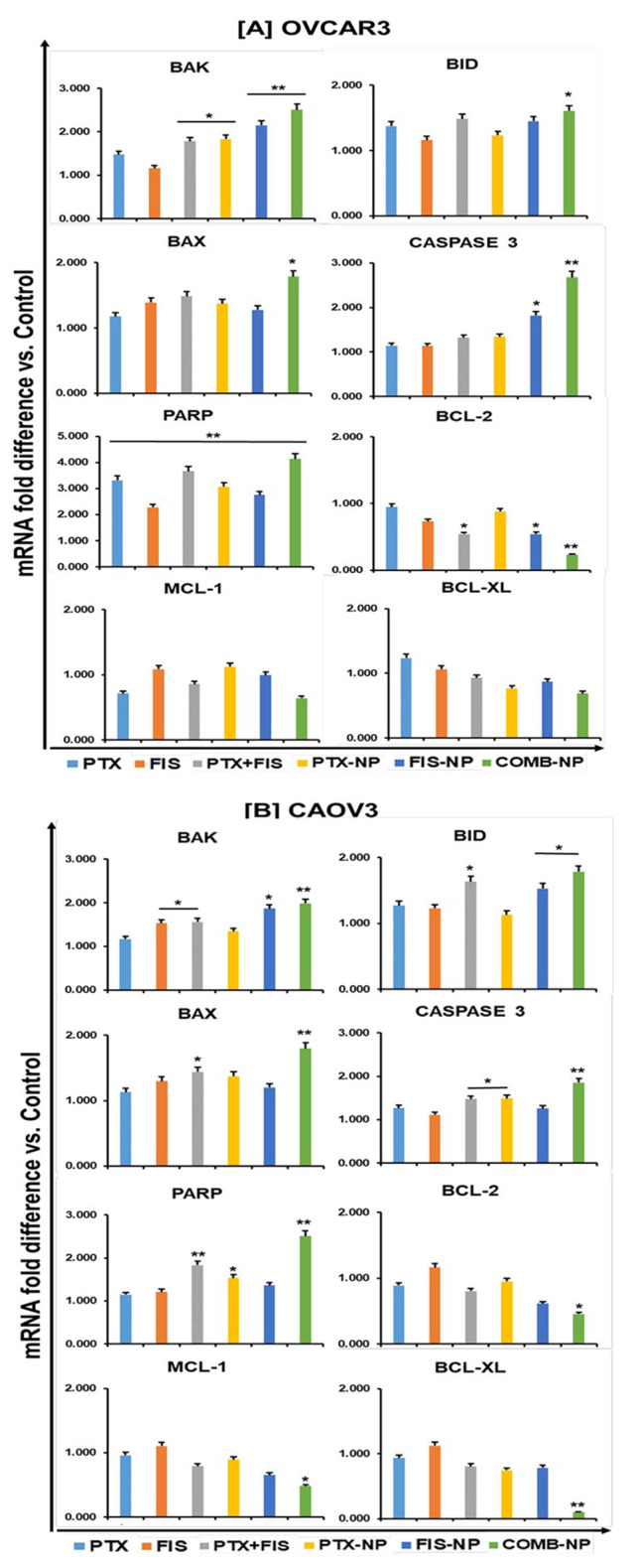
Validation of apoptosis genes in OvCa cells. mRNA expression levels of apoptotic genes were quantified by RT-PCR upon treatment and compared to controls in **[A]** OVCAR3 and **[B]** CAOV3 cells. The expression levels of caspase-3, PARP, pro-apoptotic genes (BAK, BID, and BAX), and anti-apoptotic genes (BCL-2, MCL-1, and BCL-XL) are represented by fold change relative to the control. Data were normalized to the levels of the housekeeping gene 18 S. Data are presented as the means ± standard error of the mean, and asterisks indicate significance determined by Student t-test (*P < 0.05) (**P < 0.01)

To validate these findings, RT-PCR was conducted for OvCa cells. Figure 5 shows an upregulation of BAK, caspase-3, BAX, BID, and poly-ADP ribose polymerase (PARP) in both cell lines after treatment with combination NPs, compared to the control. However, in CAOV3, there was, after treatment, a difference in downregulation of the anti-apoptotic marker BCL-XL, BCL-2, and MCL-1. While in treated OVCAR3 cells, only BCL-2 was significantly downregulated compared to the control.

### PBM NPs downregulate the MDR gene in OvCa cells

**Fig. 6:**
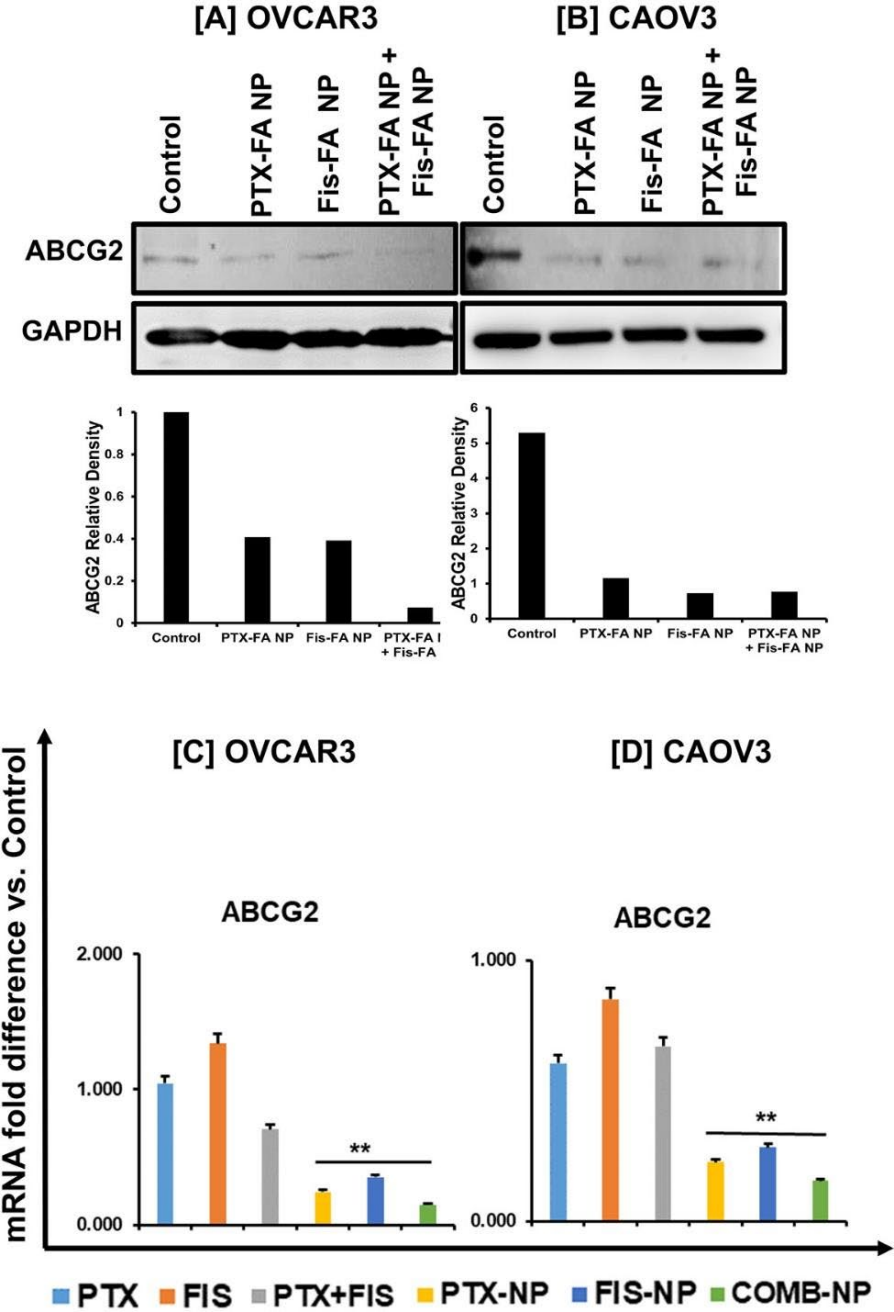
PBM NPs downregulate an MDR gene in OvCa cells. Expression of ABCG2 protein in **[A]** OVCAR3 and **[B]** CAOV3 cells treated with Fis-FA- or PTX-FA-NPs, alone or in combination for 48 h. As an internal standard for equal loading, blots were probed with a GAPDH antibody. The band intensity of ABCG2 was normalized against GAPDH, and the densitometry is shown below. mRNA expression levels of the drug resistance gene were quantified by RT-PCR upon treatment and compared to the control in both **[C]** OVCAR3 and **[D]** CAOV3 cell lines. The expression level of ABCG2 is represented by fold change relative to the control. Data were normalized to the housekeeping gene levels, 18 S expression. Data are presented as the means ± standard error of the mean, and asterisks indicate significance determined by student t-test (*P < 0.05) (**P < 0.01)

Multidrug resistance (MDR) is prevalent in platinum-resistant OvCa; it allows cells to survive exposure to anti-cancer drugs. The MDR to chemotherapeutic drugs in cancer cells is mediated through a mechanism involving P-glycoprotein (P-gp, or MDR1), multidrug resistance-associated protein 1 (MRP1 or ABCC1), or breast cancer resistance protein (BCRP or ABCG2) [23, 24]. As displayed in Fig. 6, the results show the downregulation of ABCG2 in OvCa cells upon treatment compared to the control. Additionally, RT-PCR validated the downregulation of ABCG2 after treatment with PTX-FA and Fis-FA NPs alone and in combination. These data suggest that these NPs downregulate MDR genes, reversing drug resistance in OvCa cells.

## Discussion

Among women in America, OvCa is the deadliest cancer, leaving more than two-thirds of the women diagnosed with HGSC to die [25]. This is in correlation with having no formal screening method for OvCa that can detect precancerous cells or even early-stage cancer [26]. Thus, women are generally diagnosed at advanced stages and have a 5-year survival rate of less than 50%. The standard treatment for advanced-stage OvCa patients is primary reductive surgery, followed by platinum-based chemotherapy. However, platinum resistance is high, followed by relapse in women with HGSC due to acquired MDR. Recurrence after treatment and MDR to current chemotherapeutic drugs is a problem that must be resolved [27].

PTX shows substantial effects in the treatment of advanced OvCa. However, its effectiveness has been limited due to its sometimes fatal toxicity and development of platinum resistance in OvCa cells. Consequently, synergistic treatments are required to overcome the acquired drug resistance and toxicity. Fis is a natural, non-toxic agent that has anti-cancer potential. We have found that PTX and Fis have similar mechanisms of action [28]. Our study supports the synergistic effects of PTX and Fis by examining their impact on apoptosis in OvCa cells. The results show that the combined treatment of Fis and PTX increases cells in early apoptosis compared to Fis and PTX treatments alone.

Nonetheless, treatments with free drugs affect both normal and cancerous cells. However, a carrier transporting these drugs directly to cancer cells helps ensure effectiveness and increases bioavailability due to quick absorption and metabolism [29]. We solved these issues using our distinctive, receptor-based targeted delivery approach: a PBM NP coated with FA and a PCL-PEG copolymer. FRα is overexpressed on the plasma membranes of platinum-resistant OvCas [30, 31]. Our study evaluated various ovarian tissue samples to ensure FRα expression. FRα was overexpressed in Stage 4 OvCa tissues compared to normal ovarian tissues. In addition, we examined numerous OvCa cells and found that OVCAR3 and CAOV3 cells had higher expressions of FRα in contrast to IOSE(normal), TOV-112D, and SW626 cells. As a result, OVCAR3 and CAOV3-resistant OvCa cells were selected for targeted delivery of FA-conjugated NPs. These PBM NPs coated with PCL-PEG copolymers allow enhanced bioavailability and sustained release of the encapsulated drugs. This is supported by cell culture studies showing that the PCL-PEG copolymer exhibited higher hydrophilicity and degradability than the PCL polymer alone [32]. In this regard, we generated FA- and PCL-PEG PBM NPs of PTX and Fis individually or in combination. To ensure that our NPs were formulated correctly, we looked at the physical properties by NMR. Our results are shown in the 1 H NMR spectra. These characteristics provide accurate formulations and promise effective drug delivery.

Multidrug resistance (MDR) is ubiquitous in HGSC ; it allows cells to survive treatment with anti-cancer drugs. Patients treated with platinum drugs develop platinum resistance due to the dysregulation of drug influx and efflux pathways that control the transport of drugs into cells [33, 34]. Specifically, for a range of resistant cancer cells, the ATP-binding cassette efflux transporter G2 (ABCG2) is involved in drug efflux [35]. Until now, only a few studies have looked at the capacity of Fis to enhance the anticancer activity of classical chemotherapeutic agents, such as PTX. Furthermore, its effectiveness when encapsulated in an NP that directly targets cancer cells has not been evaluated. Our results show the role of Fis in combination with PTX when encapsulated in FA-PCL-PEG-coated PBM NPs, which induced more extensive apoptosis in resistant OvCa cells compared to the cells treated with Fis or PTX alone. In addition, our study showed that Fis and PTX inhibit ABCG2 drug efflux in cancer cells, ultimately reversing the MDR that is evident after initial chemotherapy treatments.

**Fig. 7:**
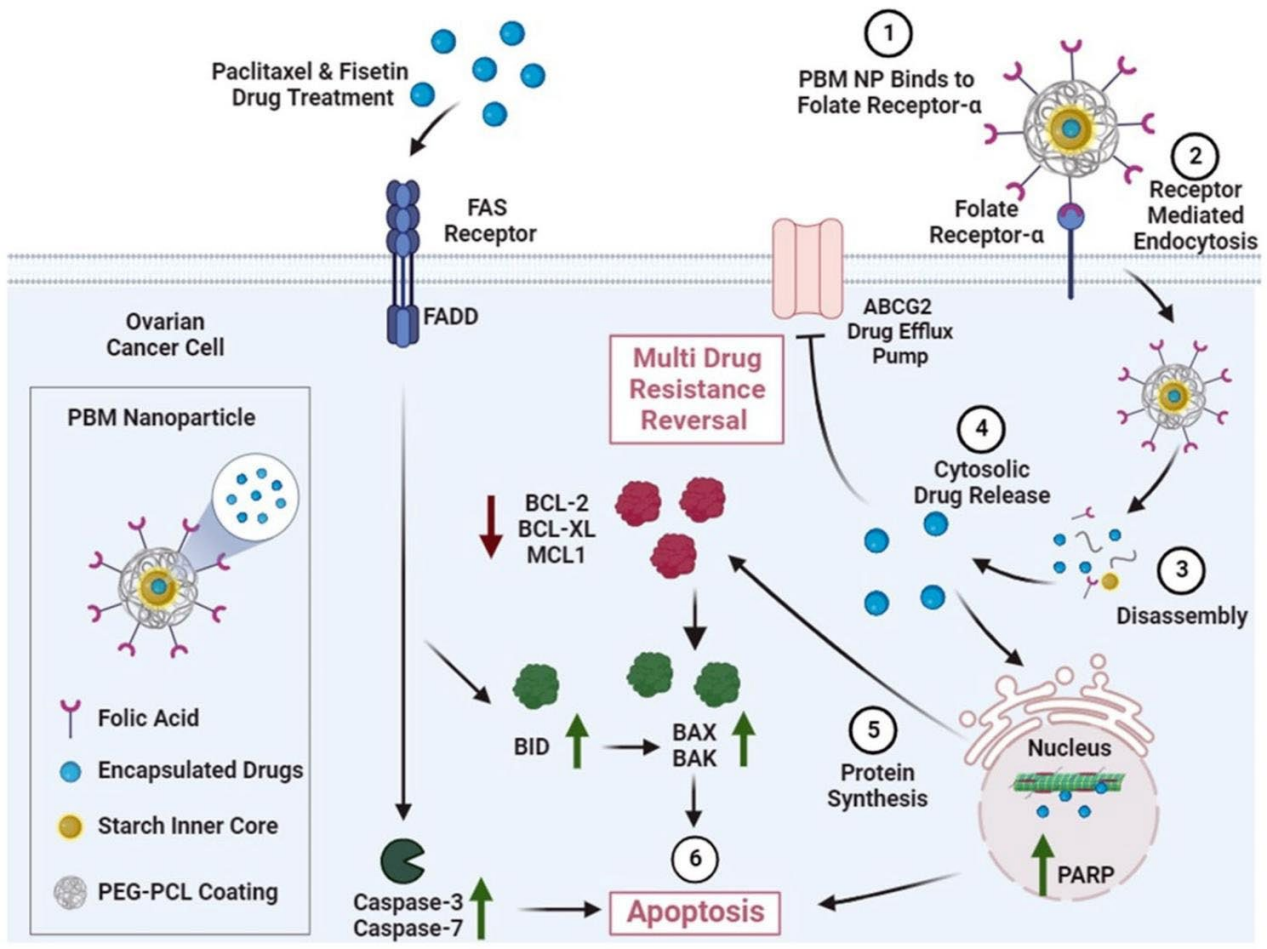
FA-PBM NPs mechanism to induce apoptosis and inhibit drug resistance in OvCa. This figure summarizes the mechanisms of combination FA-PBM NPs to induce apoptosis and inhibit drug resistance in OvCa cells. Following treatment with FA-PBM NPs, the intrinsic apoptosis pathway is initiated, triggering a cascade of events leading to the synthesis of Bcl-2 family proteins, including pro-apoptotic and anti-apoptotic proteins. The anti-apoptotic proteins, BCL-2, BCL-XL, and MCL1, are downregulated, and the pro-apoptotic proteins, BAX, BAK, and BID, are upregulated, ultimately inducing apoptosis in OvCa cells. Additionally, cytosolic Fis and PTX inhibit the drug efflux pump, ABCG2, reversing multi-drug resistance. Lastly, the anticancer drug could induce the Fas/FasL pathway to activate caspase 3 by proteolytic cleavage or stimulate BID, leading to apoptosis of OvCa cells. This evidence provides insight into how to treat platinum-resistant cancer cells that usually evade apoptosis

The mechanisms of our combination of PBM NPs to induce apoptosis and inhibit drug resistance in OvCa cells are shown in Fig. 7. Apoptosis can be initiated via two pathways: through death receptors (extrinsic) or involvement of mitochondria (intrinsic) [36, 37]. The anticancer drug-induced Fas/FasL ligand binds to intracellular Fas death domains (FADDs) and recruits pro-caspase 8 or 10 [37]. Caspase 8 then activates caspase 3 by proteolytic cleavage or stimulates BID. Our study confirms this process by showing the upregulation of caspase 3 and BID after treatment with the anti-cancer drugs PTX and Fis, which induce apoptosis in OvCa cells.

Furthermore, the intrinsic apoptosis pathway was initiated following treatment with PBM NPs. Triggering a cascade of events leads to synthesizing Bcl-2 family proteins, including pro- and anti-apoptotic proteins. The anti-apoptotic proteins within the Bcl-2 family include BCL-2, BCL-XL, and MCL1. The pro-apoptotic proteins include BAX, BAK, and BID. Our study showed that these anti-apoptotic proteins were downregulated, allowing pro-apoptotic proteins to be upregulated, leading to apoptosis of OvCa cells. This evidence provides insight into how to treat platinum-resistant cancer cells that usually evade apoptosis.

## Conclusion

In conclusion, the present study shows that FRα is overexpressed in Stage 4 OvCa tissues compared to normal ovarian tissues. Likewise, in resistant OvCa cells, FRα is overexpressed compared to normal ovarian cells. This demonstrates that the FA-conjugated PTX- and Fis-encapsulated PBM NPs target platinum-resistant OvCa cells, inducing cytotoxic/apoptotic effects and reversing MDR by regulating molecular signaling pathways. These findings allow us to create new clinical applications using PTX- and Fis-combination NPs for drug-resistant OvCa.

## Methods

### Cell lines

Human OvCa cell lines, OVCAR-3, CAOV3, TOV-112D, and SW626, were acquired from the American Tissue Culture Collection (ATCC, Manassas, VA, USA). IOSE cell line was gifted from Dr. John McDonald, Georgia Tech University, Atlanta, GA, USA. OVCAR3, IOSE, and TOV-112D cells were grown in RPMI-1640 medium supplemented with 10% fetal bovine serum (FBS), 1% HEPES, 1% nonessential amino acids, and 1% penicillin/streptomycin solution (Fisher Scientific, Pittsburgh, PA). SW626 cells were grown in L15 medium supplemented with 10% fetal bovine serum (FBS) and 1% penicillin/streptomycin solution (Fisher Scientific, Pittsburgh, PA) in non-vented flasks. CAOV3 cells were grown in Dulbecco’s Modified Eagle’s Medium (DMEM) supplemented with 15% fetal bovine serum (FBS), 1% sodium pyruvate, 1% sodium bicarbonate, and 1% penicillin/streptomycin solution (Fisher Scientific, Pittsburgh, PA). All cell lines were maintained in a standard incubator at 37 °C and 5% CO_2_.

### Materials and reagents

PTX, Fis, dimethyl sulfoxide (DMSO), soluble starch, diethyl ether, acetone, 4-(dimethylamino) pyridine, and dicyclohexylcarbodiimide were purchased from Fisher Scientific (Pittsburgh, PA). FA, N-hydroxysuccinimide (NHS), triethyl amine, polyethylene glycol (PEG), dioxane, N, N-disuccinimidyl carbonate (DSC), and polycaprolactone (PCL) were purchased from Sigma (St. Louis, MO).

### Planetary ball milling (PBM) NP formulation

The PBM method requires a milling jar holding heat-absorbent zirconium oxide planetary milling balls. The jar rotates about its axis and in the opposite direction around a common axis of the chambered wheel. This produces the rotation of planetary balls inside the jar and the milling of particles from the macroparticles containing starch (4%), polyethylene glycol, and drugs. Controlling the centrifugal force by varying the revolutions/sec (Ω), jar velocity, the size and number of the zirconium oxide balls, duration, and number of cycles, control the size of particles [12]. Fis and PTX encapsulated PBM-NPs were synthesized by adding NHS-ester-activated FA (NHS-FA) and NHS-activated FA-conjugated PEG.

### Functionalization and characterization of PBM NPs

The Singh et al. [12] protocol prepared these compounds and functionalized the PBM-NPs. To ensure accurate formulations, the following characteristics were analyzed for each PBM NP: size, zeta potential, and properties. The size and zeta potential of the Fis- and PTX- encapsulated PBM-NPs FA-PCL-PEG coated and uncoated was measured using a Malvern Zetasizer at pH 6.8 and a concentration of 0.1 mg/mL (5% mass, assuming a density of 1 g/cm^3^) of NPs. Nuclear magnetic resonance (NMR) spectroscopy was used to analyze the physical properties of PBM NPs. This allowed for identifying and quantifying the components used to formulate the NPs. A Bruker 400 MHz spectrometer was used to record the spectra.

### Immunohistochemistry

To investigate the expression of FRα, human tissue microarray (TMA) slides were acquired from US BIOMAX, Inc. (Derwood, MD). Briefly, paraffin-embedded tissue sections were deparaffinized in xylene and rehydrated through a graded alcohol series (100%, 95%, and 70%). Further, they were washed with deionized water and phosphate-buffered saline (PBS) for 5 min, followed by antigen retrieval to enhance immunogenicity and epitope availability. Subsequently, sections were washed with PBS-Tween 20, blocked with H_2_O_2_, and stained with FRα primary antibody (R&D Biosystem, Minneapolis, MN) overnight, followed by a secondary antibody. Finally, the tissue sections were developed in DAB coloring agent, counterstained with hematoxylin, dehydrated, and mounted. The images were captured and analyzed using the Aperio Scan Scope scanning system (Aperio Technologies, USA).

### Cell toxicity and viability assay

To determine cell viability, MTT (3-(4,5-dimethylthiazol-2yl)2,5-diphenyltetrazolium bromide) (Sigma, St. Louis, MO) was used. First, 10,000 OvCa cells per well were seeded in a 96-well plate. After 24 h, cells were treated with various concentrations of PTX (0.1nM – 100nM) and Fis (1µM − 100µM), alone or in combination. The cells were then incubated for 24, 48, or 72 h at 5% CO_2_ and 37 °C. Next, MTT (5 mg/mL) was added to each well, and the preparations were incubated for 2–3 h. Once the purple formazan crystals appeared, they were dissolved in DMSO (100 µL), and the absorbance was measured at 570 nm using a spectrophotometer. The same approach was applied to determine the viability of PBM-NP-treated OvCa cells. The IC50 (half-maximum inhibitory concentration) was then calculated for OvCa cells (OVCAR3 and CAOV3). The combination index (CI) was calculated for combination treatments using CompuSyn software.

### Apoptosis assay

To determine the effect of PTX-FA-NPs and Fis-FA-NPs, alone and in combination, on apoptosis, OVCAR3, and CAOV3 cells were grown and treated with IC50 concentrations of the respective drugs for 48 h. After treatment, cells were trypsinized with 0.25% trypsin and counted using a hemocytometer (Countess II FL, Life Technology). For OVCAR3 and CAOV3 cells, 100,000 cells were counted, centrifuged, and washed in PBS (Fisher Scientific, Pittsburgh, PA). In the dark, cells were stained with PI and FITC Annexin V (BioLegend, San Diego, CA) for 15 min. A Guava easyCyte HT (EMD Millipore, Billerica, MA) flow cytometer was used to analyze the apoptotic cells.

### Western blot analysis

OvCa cells, OVCAR3, and CAOV3 were treated as described earlier for 48 h and then harvested. Harvested cells were washed with cold PBS and then lysed with RIPA buffer. The cell lysates were centrifuged at 10,000 rpm for 10 min to collect the supernatants. The protein concentration was quantified using BCA protein assay kits (Thermo Fisher Scientific, Waltham, MA). Protein (30 µg) was loaded onto 10–12% polyacrylamide gels and run for ~ 1.5 h. The gels were transferred to a PVDF membrane, blocked with blocking buffer (5% non-fat dry milk (Biorad, USA) and TBS-T,( 0.1% Tween 20) (Fisher Scientific, Pittsburgh, PA) for an hour at room temperature, and then probed with primary antibodies overnight at 4 °C. The membrane was washed three times for 5 min and then probed with secondary antibodies at room temperature for 2 h. The primary antibodies used were for pro-apoptotic BAK, anti-apoptotic BCL-XL, apoptotic active caspase-3, and ABCG2; all were acquired from Cell Signaling Technology (MA, USA). The signals were then detected using enhanced chemiluminescence (ECL) (Thermo Fisher Scientific, Waltham) and imaged using an Image Quant LAS4000 (GE Healthcare-Biosciences, Pittsburgh, PA) instrument. GAPDH or β-actin (1:1000, Cell Signaling Technology, Danvers, MA, USA) was used as a control to ensure equal loading of proteins.

### Quantitative reverse transcription polymerase chain reaction (qRT-PCR)

To validate the effects of PTX-FA-NPs and Fis-FA-NPs, alone and in combination, on OvCa cells, RNA was extracted by the Trizol method (Invitrogen, Paisley, UK). OvCa cells were treated with IC50 concentrations of the agents for 48 h and lysed with the Trizol reagent, followed by mRNA isolation. Next, cDNA templates were synthesized using RT-qPCR reagent kits according to the manufacturer’s instructions (Biorad, Hercules, CA, USA). 18 S primers were used as an endogenous control. SYBR® Green PCR master mix reagents (Biorad, Hercules, CA, USA) and CFX-manager software (CFX96 Real-Time System, Biorad) were used to measure gene expression. All primer sequences (Table 1) were synthesized from the National Center for Biotechnology Information GeneBank database.

**Table 1:** List of primers used for real-time PCR analysis

Primer	Sequence
BAK Forward	5′- TTTACCGCCATCAGCAACCT-3′
BAK Reverse	5′-ATAGGCA TTCTCTGCCGTGG-3′
BID Forward	5′-AGCACAGTGCGGATTCTGTC-3′
BID Reverse	5′-ACCGTTGTTGACCTCACAGT-3′
BAX Forward	5′-AAACTGGT GCTCAAGGCCC-3′
BAX Reverse	5′-CTTCAGTGACTCGGCCAGG-3′
Caspase-3 Forward	5′-CTCTGGTTTTCGGTGGGTGT-3′
Caspase-3 Reverse	5′-CGCTTCCATGTATGATCTTTGGTT-3′
PARP Forward	5′-GCTTCAGCCTCCTTGCTACA-3′
PARP Reverse	5′-TTCGCCACTTCATCCACTCC-3′
BCL2 Forward	5′-GATAACGGAGGCTGGGATGC-3′
BCL2 Reverse	5′-TCACTTGTGGCCCAGATAGG-3′
MCL1 Forward	5′-AAGAGGCTGGGATGGGTT TG-3′
MCL1 Reverse	5′-CAGCAGCACATTCCTGATGC-3′
BCLXL Forward	5′-CCTAAGGCGGATTTGAATCTCT-3′
BCLXL Reverse	5′-TGGGCTCAACCAGTCCATTG-3′
ABCG2 Forward	5′-CCGCGACAGTTTCCAATGACCT-3′
ABCG2 Reverse	5′-GCCGAAGAGCTGCTGAGAACTGTA-3’
18 S Forward	5′-GGCCCTGTAATTGGAATGAGTC-3′
18 S Reverse	5′-CCAAGATCCAACTACGAGCTT-3′

### Statistical analysis

FRα expression intensities in TMAs were tested for normality using the Shapiro-Wilk test. The experimental data were compared using a two-tailed Student’s t-test and expressed as means ± standard error of the mean. Differences were considered statistically significant at p < 0.01. The efficacy of Fis-FA- and PTX-FA-encapsulated PBM NPs was tested for normality using the Shapiro-Wilk test. The experimental data were compared using a two-way ANOVA test, looking at concentration and time to assess the statistical significance of the data and expressed as means ± standard deviation. Differences were considered statistically significant at p < 0.05.

## Data Availability

All data generated or analyzed during this study are included in this published article.

## Abbreviations

HGSC: High-Grade Serous Carcinoma
OvCa: Ovarian Cancer
FRα: Folate Receptor Alpha
PBM-NPs: Planetary-Balled Milling Nanoparticles
FA: Folic Acid
PEG: Polyethylene Glycol
PCL: Polycaprolactone
NHS-FA: N-Hydroxysuccinimide ester-activated folic acid
NHS: N-Hydroxysuccinimide
MDR: Multidrug Resistance
TMA: Tissue Microarray
Fis: Fisetin
PTX: Paclitaxel
FBS: Fetal Bovine Serum
DMEM: Dulbecco’s Modified Eagle’s Medium
DMSO: Dimethyl Sulfoxide
PBS: Phosphate-buffered Saline
ECL: Enhanced Chemiluminescence
IC50: Half Maximum Inhibitory Concentration
CI: Combination Index
qRT-PCR: Quantitative Reverse Transcription Polymerase Chain Reaction
